# Antioxidant Activity of Essential Oil and Extracts of *Valeriana jatamansi* Roots

**DOI:** 10.1155/2014/614187

**Published:** 2014-04-06

**Authors:** Sakshima Thusoo, Sahil Gupta, Rasleen Sudan, Jaspreet Kour, Sahil Bhagat, Rashid Hussain, Madhulika Bhagat

**Affiliations:** School of Biotechnology, University of Jammu, Jammu 180006, India

## Abstract

*Valeriana jatamansi* is an indigenous medicinal plant used in the treatment of a number of diseases. In the present study, chemical composition of the essential oil was determined by GC-MS. Seven major components were identified in *Valeriana jatamansi* essential oil, namely, **β**-vatirenene, **β**-patchoulene, dehydroaromadendrene, **β**-gurjunene, patchoulic alcohol, **β**-guaiene, and **α**-muurolene. Methanolic, aqueous, and chloroform extracts of *Valeriana jatamansi* roots were also prepared and analyzed for their polyphenols and flavonoid content. Antioxidant activity of essential oil and different extracts of *Valeriana jatamansi* roots was determined by DPPH radical scavenging and chelation power assay. A linear correlation has been obtained by comparing the antioxidant activity and polyphenols and flavonoid content of the extracts. Results indicated that antioxidant activity of methanolic extract could be attributed to the presence of rich amount of polyphenols and flavonoid. Essential oil of *Valeriana jatamansi* roots showed moderate antioxidant activity.

## 1. Introduction

*Valeriana jatamansi *Jones syn.* V. wallichii *popularly known as Indian Valerian (Mushkibala in Hindi/Kashmiri, Suganthdhawal or Tagara in Sanskrit) belongs to family Valerianaceae [1].* Valeriana *is a major genus of the family Valerianaceae and is represented in all the temperate and subtropical areas of the world. In India, about 16 species and two subspecies have been reported [2].

*Valeriana jatamansi* is a small, perennial dwarf, hairy, rhizomatous herb having thick roots covered with fibers. The plant grows at an altitude of 1220–3000 m [1].* Valeriana jatamansi* is regarded as an aphrodisiac, antispasmodic, tranquilizer, antiseptic, expectorant, febrifuge, nerve tonic, ophthalmic, sedative, and tonic useful in hysteria, cholera, snakebite, scorpion sting, asthma, and eurosis [3]. Roots are acrid and bitter which are used as carminative, laxative and are also used for curing blood diseases, burning sensation, cholera, skin disease, throat troubles, and ulcers [3]. The therapeutic properties of the plant are attributed to a class of compounds called valepotriates. The valepotriates are a group of monoterpenoids of iridoid type having epoxy group and *β*-acetoxy isovaleric acids [4]. The root of* V. jatamansi* is a source of effective antileishmanial agent [5]. Root extract of* V. jatamansi* also exhibits larvicidal and adulticidal activity against different mosquito species [6]. The aqueous and methanolic extracts of rhizomes possess anti-inflammatory activity. This could be attributed to the high amount of flavonoids and tannins in the plant [7]. The objective of this study was to verify the phytochemicals and antioxidant potential of both essential oil and extracts of* Valeriana jatamansi *roots.

## 2. Materials and Methods 

### 2.1. Collection of Plant Material

Fresh plant material (roots) was collected from the high altitude of Patnitop in Jammu and Kashmir, India, and identified at the herbarium of the department of Botany, Jammu University. The material was shade dried and ground to a fine texture in a grinding machine.

### 2.2. Isolation of Essential Oil

Essential oil was extracted by hydrodistillation for 4 h using a Clevenger-type apparatus. The oil was stored at 4°C in the dark until analyzed.

### 2.3. Preparation of Extracts

The finely powdered roots were extracted with three different solvents on the basis of their polarity, that is, from nonpolar to polar. Chloroform, methanol, and water were used as solvents. 15 g of the given powdered plant material was mixed in 75 mL of each solvent and the mixture was stirred for 24 hours. The suspended mixture was filtered through whatman's filter paper and filtrate was collected. This procedure was performed thrice to get three filtrates and residue. The filtrates were then dried at room temperature. Gummy solid thus obtained after evaporation of each of the solvents was labelled and stored for further use.

### 2.4. GC-MS Analysis of Essential Oil

Analysis of the oil using gas chromatography and mass spectrometry was carried out at Indian Institute of Integrative Medicine (CSIR, India), Canal Road, Jammu, India. GC-MS 4000 (Varian, USA) system with a HP-5MS agilent column (30 m × 0.25 mm i.d., 0.25 *μ* film thickness) was used for analysis. Injector temperature was 280°C. Oven temperature programme used was holding at 50°C for 5 min, heating to 280°C at 3°C/min, and keeping the temperature constant at 280°C for 7 min. Helium was used as a carrier gas at a constant flow of 1.0 mL/min and an injection volume of 0.20 *μ*L was employed.

The MS scan parameters included electron impact ionization voltage of 70 eV, a mass range of 40–500 m/z. The identification of components of the essential oil was based on comparison of their mass spectra with those stored in NIST05 library or with mass spectra from literature [8].

### 2.5. Determination of Total Phenols and Flavonoids in Extracts

Total phenolic content was determined according to Folin-Ciocalteu method [9]. 0.5 mL of extract solution was mixed with 0.5 mL of 1 N Folin-Ciocalteu reagent. The mixture was kept for 5 min, followed by the addition of 1 mL of 20% Na_2_CO_3_. After 10 min of incubation at room temperature, the absorbance was measured at 750 nm using a spectrophotometer. The concentration of phenolic compounds was calculated according to the following equation obtained from the standard gallic acid:

(1) $$\begin{array}{c} \text{Absorbance}=0.0364\;\text{gallic}\;\text{acid}\;\left(\mu \text{g}\right)+0.009. \end{array}$$

Flavonoid content in the extract/fractions was determined by a colorimetric method [10]. Plant extracts were diluted with distilled water to a volume of 3.5 mL and 150 *μ*L of a 5% NaNO_2_ solution. After 5 min, 300 *μ*L of 10% AlCl_3_·H_2_O solution was added. After 6 min, 300 *μ*L of 1 M NaOH and 550 *μ*L of distilled water were added to prepare the mixture. The solution was mixed well and the absorbance was observed at 510 nm using UV-VIS spectrophotometer. The concentration of flavonoid compounds was calculated according to the following equation obtained from the standard quercetin graph:

(2) $$\begin{array}{c} \text{Absorbance}=0.001\;\text{quercetin}\;\left(\mu \text{g}\right)+0.032. \end{array}$$

### 2.6. DPPH Radical Scavenging Assay

The radical scavenging activity of extracts was determined with slight modifications in the method [11]. 1 mL from a 0.5 mM methanol solution of the DPPH radical was mixed to 2.0 mL sample and to this 2.0 mL of 0.1 M sodium acetate buffer (pH 5.5) was added. The mixtures were well shaken and kept at room temperature in the dark for 30 min. The absorbance was measured at 517 nm using a double beam UV-VIS spectrophotometer. Methanol was used as a negative control.

The radical scavenging activity of essential oil was determined [12]. 1 mL of different concentrations of the essential oil or bioactive fraction was mixed with 1 mL of a 90 *μ*M DPPH solution in methanol, and final volume was made to 4 mL with methanol. The mixtures were well shaken and kept at 25°C in the dark for 1 h. The absorbance was measured at 517 nm. Oil concentration providing 50% inhibition (IC_50_) was calculated from the graph by plotting inhibition% against oil concentration. BHT was used as reference.

### 2.7. Chelating Power on Ferrous (Fe^2+^) Ions

The chelating effect on ferrous ions of* Valeriana jatamansi *extracts was estimated by slight modifications in the method [13]. Different dilutions of extract/fractions were made to a volume of 3 mL with methanol. 60 *μ*L of 2 mM FeCl_2_ was added. The reaction was initiated by the addition of 120 *μ*L of 5 mM ferrozine into the mixture, which was then left at room temperature for 10 min before determining the absorbance of the mixture at 562 nm. The ratio of inhibition of ferrozine-Fe^2+^ complex formation was calculated using the following equation:

(3) %  inhibition=([absorbance  of  control−absorbance  of  test  sample]absorbance  of  control) ×100.

### 2.8. Statistical Analysis

For all the experiments, three samples were analysed and all the assays were carried out in triplicates. The results were expressed as mean values with standard deviation.

## 3. Results and Discussions

### 3.1. GC-MS Analysis of Essential Oil

Hydrodistillation of roots of* Valeriana jatamansi* produced greenish yellow essential oil with the yield of 0.8%. Chemical composition of the essential oil was determined by gas chromatography and mass spectrometry (Table 1). Seven major components were identified in essential oil* viz*., *β*-vatirenene, *β*-patchoulene, Dehydroaromadendrene, *β*-gurjunene, patchoulic alcohol, *β*-guaiene and *α*-muurolene. The chemical analysis of essential oil shows that* V. jatamansi* roots contain only sesquiterpenes in its essential oil.

### 3.2. Total Phenols and Flavonoids

Phenolic compounds have been reported to exhibit various biological activities like, antioxidant, antimicrobial etc. Total phenolic compounds in extracts were determined by Folin-Ciocalteu method and expressed as Gallic acid equivalents (GAEs). As shown in Table 2, highest amount of phenolic compound was observed in methanolic extract (187.13 ± 6.8 mg GAEs/g), followed by aqueous extract (77.66 ± 2.1 mg GAEs/g). Least amount of phenolic compounds was observed in chloroform extract (9.89 ± 0.3 mg GAEs/g). Many studies have revealed that the phenolic contents in the plants are associated with their antioxidant activities probably due to their redox properties, which allow them to act as reducing agents, hydrogen donors, and singlet oxygen quenchers [9, 14].

Total amount of flavonoids was expressed in quercetin equivalent (QE). Aqueous extract of* Valeriana jatamansi* had relatively high amount of flavonoids (452.30 ± 12.4 mg QEs/g) followed by methanolic extract (257.69 ± 9.8 mg QEs/g) and very less amount was observed in chloroform extract (5.38 ± 0.3 mg QEs/g).

### 3.3. DPPH Radical Scavenging Activity

DPPH assay has been extensively used for screening plant extracts because many samples can be accommodated in short period and are sensitive enough to detect active ingredients at low concentrations [15]. The antioxidant activity of* Valeriana jatamansi* roots essential oil and extracts is summarized in Table 3. Among all the test samples, methanolic extract of* Valeriana jatamansi* was found to be the most potent antioxidant (IC_50_ 78 ± 2.9 *μ*g/mL), followed by aqueous extract (IC_50_ values 154 ± 4.6 *μ*g/mL). Essential oil of* Valeriana jatamansi* roots showed poor radical scavenging activity (IC_50_ values 876 ± 12.8 *μ*g/mL), whereas chloroform extract showed negligible activity. BHT was taken as reference antioxidant (IC_50_ 28 ± 0.8 *μ*g/mL). A linear correlation has been obtained by comparing the antioxidant activity and polyphenols and flavonoid content of the extracts. The extracts containing good amount of phenols and flavonoids possess potential antioxidant activity. Previous studies have also reported positive correlation between phenolic and flavonoid content and DPPH radical scavenging activity of plant extracts [16]. The moderate antioxidant activity of essential oil could be attributed to the presence of sesquiterpenes.

### 3.4. Chelation Power

Chelation activity is also one of the important mechanisms of antioxidant activity. Result of chelation activity of the essential oil and different root extracts is shown in Table 3. It is evident by the data that the methanolic extract of* Valeriana jatamansi* possesses good chelation activity (76%) followed by aqueous extracts (43%) and essential oil (31%) at 100 *μ*g/mL concentration. Chloroform extract showed poor chelation activity (12%).

## 4. Conclusions

Methanol extract of roots of* Valeriana jatamansi* possesses remarkable antioxidant activity as compared to its essential oil. Thus root extract of* V. jatamansi* can prove beneficial in food and pharmaceutical industry.

## Figures and Tables

**Table 1:** Chemical composition of essential oil of *Valeriana jatamansi* roots analysed by GC-MS.

Compounds	Nature of compound	Amount in % age
*β*-patchoulene	Sesquiterpene	20.18
*β*-gurjunene	Sesquiterpene	13.0
*β*-vatirenene	Sesquiterpene	28.07
*α*-muurolene	Sesquiterpene	5.20
*β*-guaiene	Sesquiterpene	5.88
Dehydroaromadendrene	Sesquiterpene	15.92
Patchoulic alcohol	Sesquiterpene	11.72

**Table 2:** Showing total phenol and flavonoid content of different extracts of *Valeriana jatamansi* roots.

S. no.	Test sample	Total phenols (mg GAEs/g dry wt.)	Total flavonoids (mg QEs/g dry wt.)
1	Methanolic extract	187.13 ± 6.8	257.69 ± 9.8
2	Aqueous extract	77.66 ± 2.1	452.30 ± 12.4
3	Chloroform extract	9.89 ± 0.3	25.38 ± 2.0

Data is represented as mean ± SD of three triplicate experiments.

**Table 3:** Showing the antioxidant activity of the essential oil and different extracts of *Valeriana jatamansi* roots.

S. no.	Test sample	DPPH activity (IC_50 _in *μ*g/mL)	Chelation power on ferrous ions (% age at 100 *μ*g)
1	Essential oil	876 ± 12.8 *μ*g/mL	31%
2	Methanolic extract	78 ± 2.9 *μ*g/mL	76%
3	Aqueous extract	154 ± 4.6 *μ*g/mL	43%
4	Chloroform extract	—	12%
5	BHT	28 ± 0.8 *μ*g/mL	—

Data is represented as mean ± SD of three triplicate experiments.
